# DNA Libraries for the Construction of Phage Libraries: Statistical and Structural Requirements and Synthetic Methods

**DOI:** 10.3390/molecules16021625

**Published:** 2011-02-15

**Authors:** Thomas Lindner, Harald Kolmar, Uwe Haberkorn, Walter Mier

**Affiliations:** 1 Department of Nuclear Medicine, University Hospital Heidelberg, Im Neuenheimer Feld 400, D-69120 Heidelberg, Germany; 2 Institute for Organic Chemistry and Biochemistry, Darmstadt University of Technology, Petersenstrase 22, 64287 Darmstadt, Germany

**Keywords:** phage display, peptides, DNA synthesis, phage vectors

## Abstract

Peptide-based molecular probes identified by bacteriophage (phage) display technology expand the peptide repertoire for *in vivo* diagnosis and therapy of cancer. Numerous peptides that bind cancer-associated antigens have been discovered by panning phage libraries. However, until now only few of the peptides selected by phage display have entered clinical applications. The success of phage derived peptides essentially depends on the quality of the library screened. This review summarizes the methods to achieve highly homogenous libraries that cover a maximal sequence space. Biochemical and chemical strategies for the synthesis of DNA libraries and the techniques for their integration into the viral genome are discussed in detail. A focus is set on the methods that enable the exclusion of disturbing sequences. In addition, the parameters that define the variability, the minimal numbers of copies per library and the use of alternating panning cycles to avoid the loss of selected hits are evaluated.

## 1. Peptides Presented on Phages

Peptides have been proven as valuable tools in tumor diagnostic and radiotherapy. By specifically binding to receptors or other structures expressed on the surface of tumor cells, peptides are able to shuttle therapeutic drugs or radionuclides into the cells [1]. Being accessible by solid phase synthesis [2,3,4], peptides are efficient tools for diagnosis [5], therapy [6] and prevention [7] of many other diseases besides cancer.

Peptide ligands for the somatostatin or integrin receptor families are the prime examples that have been extensively optimized to enable a high tissue selectivity [8,9,10,11]. In order to extend the area of utilization of peptides for targeting purposes, ligands that bind yet unexploited receptors constitute promising candidates for the development of drugs and diagnostics.

Introduced in 1985 by George P. Smith, the phage display technology allows the screening of a vast amount of different peptides [12]. The phage display technique has been utilized for a multitude of applications [13,14,15,16], in particular as a tool for anti-cancer research. The availability of commercial libraries has further accelerated the discovery of *de novo* peptide sequences [17,18]. In this process, phages that carry a peptide that is able to interact with an immobilized target molecule are enriched via target binding and removal of non-binders by washing. Those phages that remain target-bound in the panning cycle are used to re-infect *E. coli* cells. The resulting enriched population can be used for further rounds of panning until a population of phages emerges that present target binding peptides on their surface. Determination of the DNA sequence of individual phage clones allows one to deduce the amino acid sequence of its affiliated peptide. Phage display uses the natural L-amino acids and offers a fast and convenient method for high throughput screening. Alternative peptide screening protocols that rely on genotype-phenotype coupling *in vitro* (ribosome display [19,20,21]) or *in vivo* (microbial surface display [22,23,24] have been extensively reviewed elsewhere.

The filamentous phage M13 is the most commonly used host for peptide engineering by phage display, common alternatives are the closely related phage fd [25,26] or the lytic phage λ [27]. The preference of M13 is the result of the commercial availability of its engineered vectors and ready-to-use libraries, for example those offered by New England Biolabs, Inc. Furthermore its straightforward manipulation and the comprehensive understanding of the viral life cycle and the phage structure contribute to its popularity. Following the infection of the host, the single-stranded M13 genome is converted to its double-stranded replicative form to produce viral proteins and single-stranded DNA progeny. The viral coat proteins are anchored in the host cell membrane and form the viral particle while the single-stranded DNA is extruded through the membrane. The virion is a long and flexible rod, about 1 µm in length and 10 nm in diameter. The viral coat consists of approximately 2,700 copies of the helical major coat protein pVIII and the class of minor coat proteins, each approximately 5 copies. The minor coat proteins pIII and pVI occupy one end, while the other minor coat proteins pVII and pIX cover the opposite end. Despite producing ca. 1,000 particles per hour, the infected host survives and proliferates due to the non lytic life cycle of M13 [28].

Even though the coat protein pIII is essential for the reproduction by interacting with the pili of *E. coli*, it is the most popular target for modifications. This is due to the fact that it can carry up to 50 additional amino acids without reduced infectivity. Randomization of a stretch of nucleotides that are fused to the pIII gene allows for the construction of a phage population, with a different peptide on the surface of each individual clone [29,30]. Moreover, phagemid vectors have been established that contain the coding sequence for a full-length or shortened version of pIII with a fused peptide/protein sequence together with a phage replication origin. Upon phage infection of *E. coli*, cells carrying that vector phage particles can be produced that display both the infectious full length pIII and the plasmid-encoded modified pIII protein.

The phage T7 offers an additional established platform for phage display. The common attachment point is its minor capsid protein 10B. Libraries or kits for library construction are marketed by Calbiochem. It has a lysogenic and a lytic life cycle. The lytic life cycle can be induced and ends in the release of mature phages. While its reproduction requires a higher number of steps than the reproduction of M13, *in-vitro* encapsulation of foreign DNA enables a more economic way to introduce synthetic DNA libraries. The various advantages of T7 over M13 display techniques [31] result from the facts that the capsid is not involved in the docking steps of infection and that the assembly of the virion proceeds without migration through the cell membrane of the host.

## 2. DNA Libraries

Since 20 different natural amino acids exist, the number of different peptide sequences that can be obtained by randomizing N residues is 20^N^. Table 1 shows the number of possible variants of fully randomized peptide sequences that can be obtained by simultaneously randomizing 7 to 16 residues. The success of a phage display experiment strictly relies on the quality of the initial DNA-library, which is mainly defined by its diversity. There are two factors that limit the maximal number of different phage clones that can be obtained, namely the amount of phage-encoding DNA molecules that can be generated *in vitro* and the efficiency of their introduction into *E. coli* cells via transformation. The maximal number of different phage encoded peptides of a hexapeptide library can be estimated as follows: an equimolar mixture of the four nucleotides has an average weight of 325 g/mol, the mean weight of a triplet is approximately 920 g/mol. A randomized DNA of a hexapeptide library, for example the one constructed by Cwirla *et al.* [32], has a molecular weight of about 5,500 g/mol (15,000 g/mol including primer regions). 1 µg of this insert contains more than 10^13^ molecules (which represents a very small fraction of the 7.2 kb long single strand DNA of the phage with approximately 2.2 × 10^6^ g/mol). In its digested and modified double stranded form, 1 µg of pure vector contains about 10^11^ copies. Considering that only 1% of this DNA is transferred into *E. coli* by electroporation, about 10 µg of an engineered vector are required to obtain a library with 10^9^ individual clones, each present in 1,000 copies.

**Table 1:** Correlation between the number of random amino acids and the maximal diversity of a peptide library.

random positions	individual sequences	random positions	individual sequences
7	1 × 10^9^	12	4 × 10^15^
8	2 × 10^10^	13	1 × 10^17^
9	5 × 10^11^	14	2 × 10^18^
10	1 × 10^13^	15	3 × 10^19^
11	2 × 10^14^	16	6 × 10^20^

Table 1 shows that by applying this type of library construction, all possible sequences of a random 7-mer peptide can be covered. However, only 1% of the sequence space of a random 9-mer peptide is approximately covered, while a 12-mer library expresses less than 0.001% of the possible individuals.

A primary task of library synthesis is to obtain an optimal distribution of these possible individuals in the sequence space. In the following sections we provide the essential knowledge to understand how the synthetic procedures used for the construction of phage libraries give access to high quality and reliable peptide pools.

### 2.1. Chemical Synthesis of Library Inserts

Based on the structural elucidation by Watson and Crick in 1953 [33,34] and the introduction of the phosphoramidite chemistry in 1981 [35], solid phase synthesis [36] is the method of choice for the synthesis of standard and random oligomeric DNA primers. When used for peptide screening, peptide-encoding DNA libraries based on partially randomized oligonucleotides have to meet many requirements as defined chain length, correct sequences vicinal to the random section, appropriate placement of primer and restriction sites and above all, the purity of the DNA pool.

**Figure 1:**
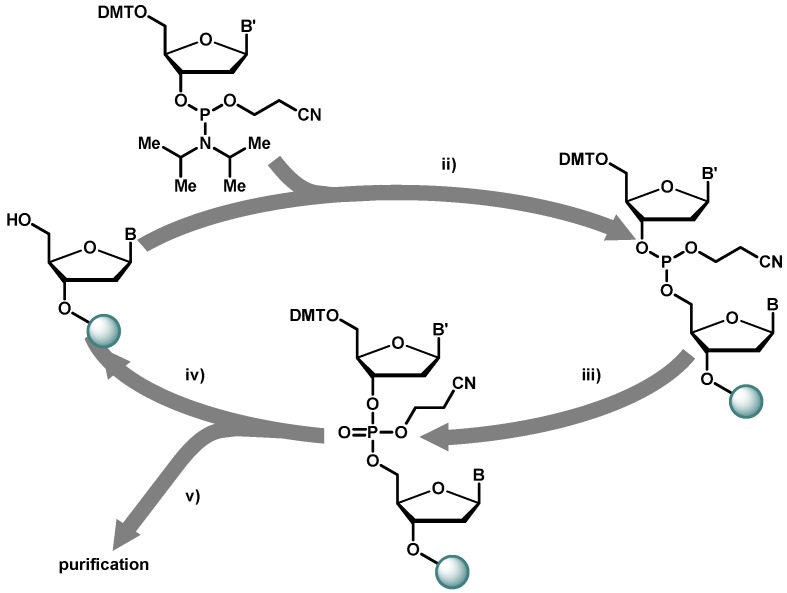
Solid phase oligonucleotide synthesis cycle. DMT = 4,4’-dimethoxytrityl;B,B’ = protected nucleobase. Key: (i) activation (e.g., with tetrazole); (ii) iodine-oxidation; (iii) trichloroacetic acid deprotection; (iv) NH_3_-cleavage.

Figure 1 shows the cycle of the automated solid phase DNA synthesis using phosphoramidite building blocks. The first nucleotide is attached via its 3’ hydroxyl function to a flexible linker on the solid support, controlled pore glass. Long spacers and low degrees of loading of the solid support reduce the amount of side products formed and improve the yields of oligonucleotide synthesis. After trichloroacetic acid deprotection of the first dimethoxytrityl moiety, the free 5’-hydroxy function is reacted with an activated nucleotide. Phosphoramidites are in the oxidation state +3, in order to obtain phosphates; the phosphorus in the coupling product has to be oxidized by iodine to obtain oxidation state +5 prior to deprotection. These three steps, coupling, oxidation and deprotection are repeated for every nucleotide to obtain the desired sequence.

The final oligonucleotide is cleaved from the solid support by concentrated ammonia to remove the remaining β-cyanoethyl protecting groups from the phosphate backbone and to liberate the nucleobases from their protection groups. The DMT protection of the last base is split off after purification to improve the separation from capped sequences. The DMT protection group acts as a lipophilic anchor in the reversed phase HPLC purification process [37]. For biochemical applications the 5’-hydroxy function can be phosphorylated using a kinase. With modern fully automated oligonucleotide synthesizers, coupling yields up to 99.5% per synthesis step can be achieved making the synthesis of oligonucleotides longer than 100 bases feasible.

### 2.2. Source of Variability

The most straightforward method to obtain a randomized oligonucleotide sequence is the use of a equimolar mixture of the four activated nucleotides in the coupling step. However, this strategy suffers from the fact that, as shown in Table 2, together with the 61 sense codons, three stop codons are incorporated at the randomized position into the oligonucleotide primers. The stop codons prevent the biosynthesis of the coat protein by the host and uninfective virions are produced.

**Table 2:** Trinucleotide codons and translation into amino acids.

TTT	Phe	TCT	Ser	TAT	Tyr	TGT	Cys
TTC		TCC		TAC		TGC	
TTA	Leu	TCA		TAA	**STOP**	TGA	**STOP**
TTG		TCG		TAG	**STOP**	TGG	Trp
CTT		CCT	Pro	CAT	His	CGT	Arg
CTG		CCC		CAC		CGC	
CTA		CCA		CAA	Gln	CGA	
CTG		CCG		CAG		CGG	
ATT	Ile	ACT	Thr	AAT	Asn	AGT	Ser
ATG		ACG		AAC		AGC	
ATA		ACA		AAA	Lys	AGA	Arg
ATG	Met	ACG		AAG		AGG	
GTT	Val	GCT	Ala	GAT	Asp	GGT	Gly
GTC		GCC		GAC		GGC	
GTA		GCA		GAA	Glu	GGA	
GTG		GCG		GAG		GGG	

The formation of homogenously distributing sequences is enhanced by incorporating only a mixture of guanine and thymine or guanine and cytosine in position three of the codons. This strategy leads to the elimination of two of three stop-codons, while the remaining 32 triplets code for all 20 amino acids. The remaining stop codon TAG can be suppressed by a *sup*E *E. coli* strain used for phage propagation that contains genes for the corresponding tRNA, which eventually results in the incorporation of a glutamine residue at the position of the stop codon during translation, albeit with varying efficiency [38].

#### Improvement of statistic distribution by exclusion of rare codons

Among the 64 possible trinucleotides are several combinations, which code for one amino acid, but the relative abundance of the amino acids is not proportional to the number of its codons. Moreover, certain codons that possess suboptimal tRNA anticodon binding are avoided by *E. coli*, leading to marginal concentrations of the corresponding tRNA. Arginine, for example is represented by one major and one minor codon, while four codons are virtually unused in the highly expressed proteins of *E. coli* [39,40]. Certain rare codons are used to regulate expression, induce structural information by deceleration of protein assembly or are necessary as reading frame shifts and some others have still yet unidentified features [41,42,43,44].

In certain cases it is advantageous to limit the number of possible amino acids in the randomized positions. Various possibilities to construct limited random codon sets exist, e.g. usage of thymidine in position two and randomization of the first and third nucleotide results in the exclusive expression of isoleucine, leucine, methionine, phenylalanine and valine. Mena *et al*. developed a computational tool to design degenerate codons, providing assistance in library design [45]. Table 3 shows a selection of codon sets to narrow the degree of randomization to four amino acids, which were utilized by Fellouse *et al*. to construct Fab fragments with impressive antigen binding characteristics [46].

**Table 3:** Selected codon sets from Feelouse *et al*. [46], which limit randomization to four possible amino acids.

G/T**–**A/C**–**T	Tyr, Ala, Asp, Ser	A/G**–**G/C**–**A	Thr, Arg, Gly, Ala
A/T**–**A/C**–**T	Tyr, Thr, Asn, Ser	G/C**–**A/G**–**C	His, Arg, Asp, Gly
C/T**–**A/C**–**T	Tyr, Pro, His, Ser	A/G**–**G/C**–**T	Gly, Ala, Thr, Ser

The best method for the synthesis of defined random primers, which homogenously incorporate all amino acids, is the trinucleotide approach. In this strategy, the initial DNA-library is assembled by utilizing the trinucleotide building-blocks shown in Table 4. This eliminates the integration of stop as well as rare codons and their possible accumulation, which may cause translational problems, like frame shifting [47].

**Table 4:** List of codons recommended for the trinucleotide approach by Kayushin *et al*. [48].

Ala	GCT	Arg	CGT	Asn	AAC	Asp	GAC	Cys	TGC
Gln	CAG	Glu	GAA	Gly	GGT	His	CAT	Ile	ATC
Leu	CTG	Lys	AAA	Met	ATG	Phe	TTC	Pro	CCG
Ser	TCT	Thr	ACT	Trp	TGG	Tyr	TAC	Val	GTT

Even though expensive phosphoramidite trimer building blocks are required, this technique is the method of choice for long sequences, or protein evolution applications, based on chemical synthesis. Due to the decreased number of coupling steps the amount of side products is reduced, which facilitates purification and increases the overall yield. By this way the amino acid distribution is also improved, as e.g. shown by Krumpe *et al*. who generated a T7-phage 12-mer peptide library by the trinucleotide method and found it to possess a higher diversity than its conventionally assembled counterpart [49]. The elimination of redundant or incompatible codons and a precisely tuneable distribution of amino acids constitute further advantages.

**Scheme 1:**
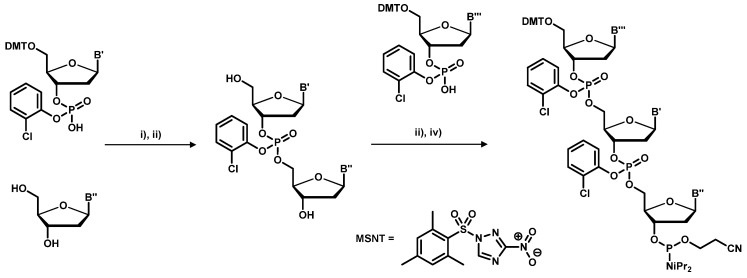
Preparation of trinucleotide building blocks. (i) MSNT-coupling; (ii) DMT-cleavage; (iii) phosphoramidite formation using a phosphordiamidite reagent.

The trinucleotide building blocks are efficiently synthesized in solution making use of the enhanced selectivity of the MSNT-activated *o*-chlorophenyl phosphordiesters, as shown in Scheme 1. Both 3’ and 5’-hydroxy functions do not require protecting groups, but the products require laborious workup following each reaction step. Introduced by Virnekäs *et al*. in 1994 [50], several techniques have been employed to enhance the outcome of the phosphoramidite synthesis [48,51]. One major problem with the use of trinucleotide building blocks are the large differences in the reactivity of the trinucleotide phosphoramidites. Hence, to obtain equal distribution of all codons at each position of a random oligonucleotide, non-equimolar mixtures of trinucleotides have to be used where more reactive trinucletoides are present at reduced concentration and vice versa [48,51].

Other DNA synthesis strategies were developed that encode random amino acids using a subset of orthogonally protected tri- and dinucleotide phosphoramidites [52]. An alternative is to split the resin prior to the coupling followed by treatment with four different mixtures of dinucleotides and subsequent coupling of a single nucleotide. This method allows the incorporation of all highly expressed codons as well [53].

### 2.3. Biological Synthesis of Random Library Inserts

To introduce random mutations in large proteins as e.g., antibodies or enzymes, error prone PCR or gene shuffling techniques often are the method of choice [54]. To introduce mutations in peptides, local randomization can be achieved using oligonucleotide mixtures as primers for *in vitro* DNA synthesis. The best known procedure was introduced 1978 by Michael Smith, who was honoured with the Nobel Prize in 1993 [55]. It is based on the fact that short oligonucleotide primers can bind with high sequence specificity to a DNA template and be extended by DNA-polymerases, *i.e.*, the Klenow-fragment. The polymerase can tolerate mismatches, as long as a stretch of at least 6–10 nucleotides at the 3’ end of the oligonucleotide is fully complementary to the template strand. Under optimized conditions, starting from a random oligonucleotide that is hybridized to M13 phage single stranded DNA, a full length DNA strand can be generated that contains the desired mutation. Several techniques were established to remove the unmodified template strand, the most popular being the use of a template strand that contains deoxyuridine in place of thymidine which results in its degradation in *E. coli* upon transformation [56].

### 2.4. Cloning Technique for the Integration of Oligonucleotide Sequences into the Phage Genome

**Figure 2:**
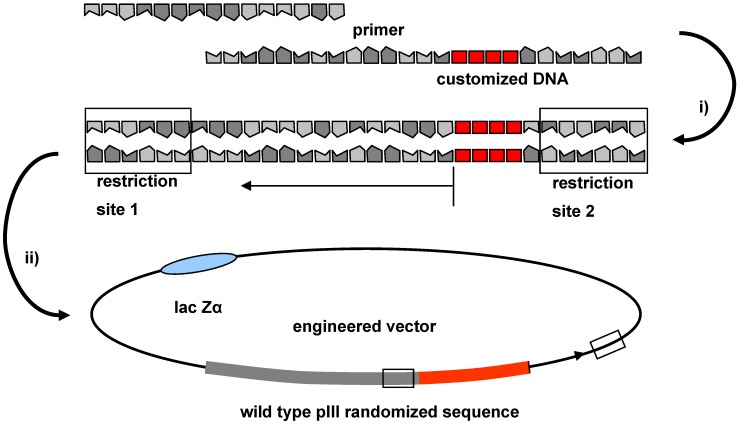
Schematic presentation of the introduction of foreign DNA; (i) annealing and PCR; (ii) separate double digestion, followed by DNA-ligation with an appropriate vector in digested form.

During recent years, alternative protocols were developed that rely on generation of double stranded DNA stretches by annealing two complementary oligonucleotides followed by fill-in reaction (Figure 2). These are designed such that they contain appropriate restriction sites at their ends which also occur at the terminus of the phage pIII gene. To achieve an optimal insertion, it is recommended to use two different restriction enzymes that produce sticky ends. Non-productive inserts, *i.e.*, primer dimers, partially digested DNAs, or incomplete linear ligation products are produced as contaminants in the PCR amplification and the ligation processes. For purification, the double stranded products are subjected to preparative gel electrophoresis (using either acryl amide or agarose gels). This separation technique discriminates side products according to their size. As a result of the randomisation the product bands are broadened. Consequently, the removal is limited to side products that significantly differ in size and structure from the products desired. Temperature or denaturing gradient as well as pulsed field gel electrophoresis offer alternatives, but are time and work demanding.

The vector and the insert usually are digested separately and purified before the fragments are annealed and fused by a DNA ligase to obtain the double stranded cyclic DNA ready for transformation by electroporation. To avoid background transformants that contain a plasmid lacking the desired DNA fragment it is highly recommended to purify the vector fragment from remaining traces of undigested or partially cleaved vector DNA after restriction enzyme cleavage. A very straightforward procedure for removal of these side products is the isolation of linear vector DNA by sucrose gradient density centrifugation [57]. Though the transformation efficiency of the electrocompetent cells is high and may approach 10^9^–10^10^ transformants per µg supercoiled plasmid DNA, in practice the yield is often much lower for ligation products and multiple parallel transformations using several hundred µg of DNA are often required to obtain >10^9^ transformants.

### 2.5. Determining the Variability of the Completed Peptide Library

After electroporation and growth, individual bacterial colonies are propagated and the produced viral material is analysed by DNA sequencing. To assure a reasonable significance, at least 50–100 phage clones should be sequenced. Factors of interest are the frequency of appearance of each amino acid and the distribution of dipeptide fragments. For example, DeGraaf *et al*. constructed a decapeptide library and 52 individual clones were examined to determine the nucleotide diversity and frequency of the amino acids. The analysis of 520 dipeptides showed that 245 dipeptides were present in this library. Considering the theoretical number of 400 possible permutations this analysis revealed that a high diversity was achieved [58]. Equations to calculate the diversity of a phage library and the RELIC database offer improved statistical analysis for this process [59,60,61]. Moreover, next-generation-sequencing offers the opportunity to check the quality of the library by sequencing tens of thousands individual clones [62]. In addition the library can be tested by isolating phages that bind well-characterized targets like immobilized streptavidin. In the case of streptavidin the bound phages, which are eluted by biotin, are expected to carry the mimotope sequence His-Pro-Gln in the displayed peptide [63].

**Figure 3:**
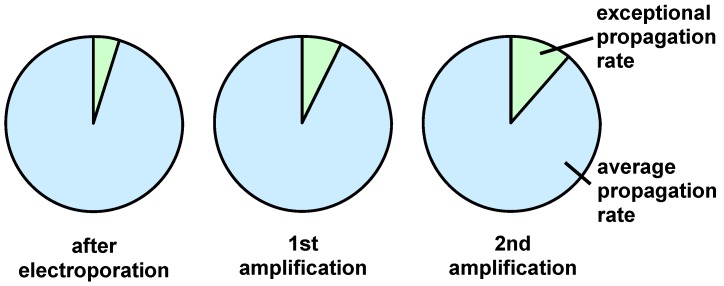
Schematic illustration of the results obtained with clones possessing different propagation rates.

After the final electroporation and diversity analysis of the gene pool it would be desirable to expand the number of phage copies of the initial phage library via re-infection of *E. coli* and generation of multiple copies of each library member. However, this procedure is at risk to compromise the initial library diversity due to non linear propagation of the individual clones. Figure 3 shows the relative clone accumulation obtained in two amplification steps, if 5 percent of the individual clones in the initial library propagate with a growth rate differing by the factor 1.5. 

This clearly causes a significant bias of the homogeneity of the library to be used in the following screening experiments.

## 3. Screening Procedures

Screening protocols differ in many aspects such as presentation of the immobilized target to the phage population or the extraction of target-bound phages [64]. The target can for example be immobilized to plastic surfaces, magnetic beads or presented on the surface of whole cells [65]. It is even possible to use tumor bearing animals for *in vivo* selection by sacrificing the animal and propagation of the phages enriched in the tumor tissue [66].

**Figure 4:**
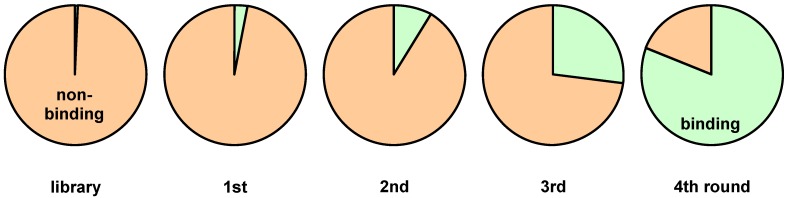
Enrichment of binding phages by affinity selection.

In an idealized panning experiment less than 1 percent of the initially used library can be expected to bind to the target prior to extraction and amplification in *E. coli*. Considering an amplification factor of three for a selectively binding clone, the library would consist of 81 percent binding individuals after four cycles as illustrated in Figure 4.

**Figure 5:**
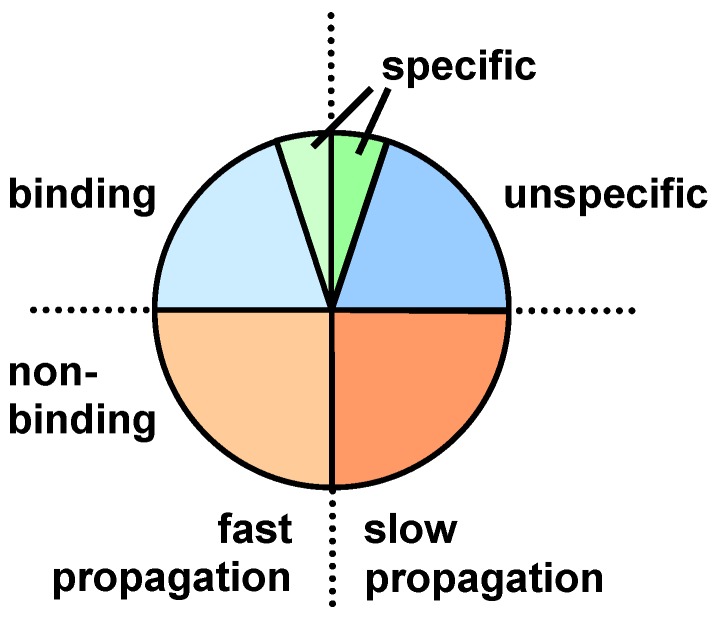
Schematic representation of the different types of phages with respect to their target binding and evolutionary fitness.

Considering that most clones present in a library bind the target by unspecific interactions and possess different evolutionary fitness, phages can be allocated to three categories: non-binding, unspecific binding and specific binding, which are subdivided into fit and unfit individuals as schematically shown in Figure 5.

**Figure 6:**
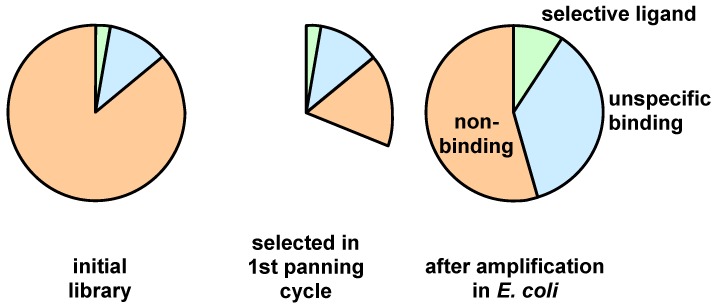
Schematic representation of the relative amplification of unspecific binders with a high propagation rate versus selective binders with moderate propagation rates after one panning round.

A serious difficulty can emerge for specific binders that are present in a low number of copies and slowly propagating. Unspecific binders with high propagation competence can outgrow the specific binders in the following amplification step. Subsequent selection by stringent competition amongst the clones or negative panning is not sufficient to remove the unspecific clones (Figure 6).

**Figure 7:**
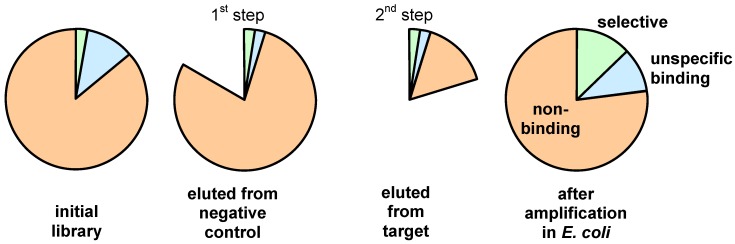
Schematic representation of the effect obtained by applying negative panning prior to the positive selection step.

This problem can be overcome by starting the panning, as shown in Figure 7, with a negative panning round. In this case the amount of the unspecifically binding phages with a high propagation rate is significantly reduced. The depletion (negative panning) can be achieved by affinity chromatography of the library using a column that contains the agent that is used to immobilize the target structure [30]. The subsequent positive panning is then performed with a population that is depleted from unselective clones.

As phage libraries with a high number of random positions contain only a small fraction of the possible peptide motifs, a mutagenesis approach can be applied for the fine tuning of the initially obtained clones [67]. This secondary library can be constructed for instance by error prone PCR or by using random primers. Despite to the low mutation rate, error prone PCR possesses the advantage that only DNA isolated from panning is required, while the primer method offers high mutation rates and directed leitmotif optimization but requires defined oligonucleotides, which can cover the whole random region.

## 4. Sequence and Structural Motifs for Displayed Peptides

Peptides that form secondary structures possess multiple advantages, for example higher stability *in vivo* and enhanced target affinity due to a decreased loss of entropy associated with their ligand-receptor binding. Phage display enables the application of a variety of such mini-protein scaffolds. The scaffolds can mimic antibodies, posses better pharmacokinetics and are accessible by synthetic chemistry [68,69,70]. Disulfide bridges are a widespread component of many natural miniproteins like scorpion-venoms or plant-toxins [71]. They are often used in phage display, because of their enhanced half life and binding characteristics [72]. One or more disulfide bridges are the most common used method to introduce constraints similar to natural compounds like antibodies or de novo cyclic peptides [73,74,75,76,77].

Chemical modification of surface peptides is a new and promising approach. The prime example for this methodology is a library containing bicyclic peptides developed by Heinis *et al*. In this library three cysteine residues at fixed positions are interconnected by 1,3,5-tris(bromomethylene)benzene. Expression of the peptide, which contains three thiol groups, required the constructing pIII gene that encodes for a cysteine free pIII protein. This chemically modified phage library enabled the selection of a plasma kallikrein inhibitor with a nanomolar inhibition constant [78]. Alpha helices and beta sheets can be constructed by coding lipophilic and hydrophilic amino acids at distinct repeats. A random lipophilic position is achieved by placing in a codon a thymidine central between two fully randomized nucleotides; the hydrophilic counterpart is coded by a C or A in the first, A in the second and a mix of every nucleotide in third position. Moreover, basic or acidic positions can be designated by choosing the codon sets A-A/G-A/G for Lys and Arg or G-A-T/C/A/G for Asp and Glu. If these two randomized codon groups are arranged in an alternating fashion, beta sheets result. A heptad repeat, *i.e.* a lipophilic amino acid in positions a, d and e, produces a bundle of four alpha helices [79].

A very promising new approach relies on the expansion of the genetic code via phage display of peptides and proteins containing unnatural amino acids. This work was pioneered by Schultz and co-workers and relies on the presence in *E. coli* of an additional orthogonal tRNA/aminoacyl-tRNA synthetase pair capable of incorporating various unnatural amino acids into proteins in response to unique nonsense codons [80,81]. More examples for phage library screening of peptides containing unnatural residues can be expected to come over the next years expanding the scope of phage display technology.

## 5. Conclusions

Most of the work on phage display screening is performed with commercially available libraries. However, the potential of these libraries is restricted – specifically designed libraries expand the scope of phage display techniques. The review discusses the biochemical and chemical strategies that allow the synthesis of homogenous libraries with an optimal coverage of their maximal diversity. Moreover, the statistical and technical considerations that allow to deduce optimal screening strategies are discussed. The consideration of several factors such as the selection of the technique to introduce the diversity and the methods of amplification of the library is mandatory to obtain reliable libraries with an optimal diversity. Besides the synthesis of the random primers and their insertion into the phage library, the selection and propagation of the specifically binding phage populations have to be taken into account.
